# Five Year Review of All Examined Corneal Tissue in a Tertiary Eye Care Center: Demographics and Surgical Indications

**DOI:** 10.1007/s44197-024-00328-z

**Published:** 2024-11-26

**Authors:** Bushra Kokandi, Saeed Al Zahrani, Hala A. Helmi, Khalid M. Alshomar, Hind Manaa Alkatan

**Affiliations:** 1 Ophthalmology Department, King Saud University, Riyadh, Saudi Arabia; 2 Jeddah Eye Hospital, Jeddah, Saudi Arabia; 3 Department of Ophthalmology, McGill University, Montreal, Canada; 4 Ophthalmology Department, King Faisal Specialist Hospital and Research Center, Riyadh, Saudi Arabia; 5 Ad Diriyah Hospital, Riyadh Third Health Cluster (Ministry of Health), Riyadh, Saudi Arabia; 6 Pathology Department, King Saud University, Riyadh, Saudi Arabia; 7 King Saud University Medical City, Riyadh, Saudi Arabia; 8 Departments of Ophthalmology & Pathology, College of Medicine, King Saud University, PO Box 18097, Riyadh, 11415 Saudi Arabia

**Keywords:** Cornea, Keratoplasty, DSAEK, Keratectomy, Biopsy, Keratoconus, Graft, Descemet’s membrane, Keratitis

## Abstract

**Background:**

Corneal pathologies are among the most common reversible sight-threatening diseases globally. As such, corneal tissue transplantation (keratoplasty) techniques are evolving over time depending on the prevalence of different pathologies in each geographical area. We aim to provide a baseline information on the common keratoplasty procedures performed in our area in relation to prevalent corneal pathologies and to analyze common corneal surgical practice trends in our area. Other types of corneal tissue sampling (superficial keratectomy and corneal biopsy) indicated for therapeutic and diagnostic purposes are also included.

**Methods:**

This is a retrospective cohort study. All corneal tissue specimens, including those harvested for both therapeutic and diagnostic purposes, received for histopathological examination at our center over a period of 5 years were collected along with the respective demographic data, clinical diagnoses, and surgical indications. Descriptive analysis was used to elucidate important conclusions, and comparative analysis was used to highlight differences between different types of keratoplasty specimens in relation to the surgical indications.

**Results:**

A total of 347 patients’ corneal tissue specimens were included. Males accounted for 52.45%. Full-thickness corneal buttons were the most common (*n* = 172), followed by partial-thickness corneal specimens (*n* = 75), and Descemet’s membrane and endothelium samples (*n* = 63). Top surgical indications for keratoplasty were keratoconus (*n* = 149), followed by bullous keratopathy (*n* = 61), failed previous keratoplasty (*n* = 47), corneal ulceration (*n* = 33) and corneal dystrophies (*n* = 22). Patients undergoing penetrating or lamellar keratoplasty were significantly younger (*p < 0.001*). Superficial keratectomy and corneal biopsy for keratitis were significantly more common among male patients (*p = 0.041*), while failed endothelial keratoplasty was observed more among females (*p = 0.026*).

**Conclusion:**

Our findings highlight the evolving landscape of corneal transplantation and the importance of tailoring surgical approaches to address the specific needs and risk profiles in different populations. Keratoconus is a leading cause for corneal grafting and seems to constitute a major treatable and visually disabling disease in Saudi Arabia, thus may require further screening and genetic studies with consideration for preventive measures.

## Introduction

In 1905, corneal transplantation emerged as a pioneering procedure with good outcomes, heralding a new era in the field of corneal surgeries [1]. Corneal transplantation became an effective modality for restoring vision in individuals afflicted with visual impairments secondary to different pathologies. The field of keratoplasty has undergone significant advancements and evolved into a dynamic discipline characterized by a diverse range of surgical interventions, each designed to address specific clinical scenarios and optimize patient outcomes.

A comprehensive analysis of a 10-year data search through PubMed, spanning from 2001 to 2015, was gathered from three distinct geographical regions including the United States [2], British Columbia in Canada [3], and the West of Scotland [4], which revealed the primary indications for keratoplasty. The leading indications were aphakic/pseudophakic bullous keratopathy (ABK)/(PBK), corneal edema, keratoconus, Fuch’s endothelial dystrophy (FED), and regrafting. Among these, penetrating keratoplasty (PKP) appeared as the most performed surgical procedure, while endothelial keratoplasty (EK) has demonstrated a rising trend since the year 2007 [2–4]. These findings underscore the evolution and shifting landscape of keratoplasty techniques. On the other hand, smaller corneal tissue specimens might be indicated for diagnostic purposes such as in cases of infectious keratitis.

Hence, our study aimed to investigate the trends and the demographic variables in relation to common keratoplasty techniques, which may reflect the learning curve among corneal surgeons in our area. The histopathological spectrum of all types of corneal tissue specimens received in our laboratory along with the corresponding clinical indications over a 5-year period were collected to reach conclusive facts using both descriptive and statistical analysis since this is being conducted in a tertiary eye care center with referrals from across the kingdom.

## Materials and Methods

This retrospective study was granted approval by the Thesis Committee in addition to the Human Ethics Committee/Institutional Review Board (HEC/IRB) at King Saud University, Riyadh, Saudi Arabia (Research Project No. E-24-9031). A general informed written consent was obtained from the patients and/or guardians of all cases which includes permission as part of the common practice for anonymous use of data for the purpose of publication.

The study included all corneal tissue specimens obtained between May 2015 and December 2019 and received in our histopathology lab for examination and tissue diagnosis. We collected a total of 347 specimens, which included tissues obtained from different keratoplasty procedures in addition to other types of corneal tissue specimens including superficial keratectomy (SK) and/or corneal biopsy. Data extraction focused on acquiring demographics information, clinical presentations, the specific type of keratoplasty performed, detailed histopathology findings, and diagnoses.

The corneal tissue specimens were categorized into the following types: keratoplasty specimens including PKP specimens (full-thickness cornea), lamellar keratoplasty (LKP) specimens (partial-thickness cornea), and EK specimens. The latter included specimens obtained from Descemet’s stripping automated endothelial keratoplasty (DSAEK) or failed DSAEK grafts. In addition, SK specimens (partial-thickness anterior corneal tissue) and corneal biopsies were also included. Patient demographics and clinical indications for each keratoplasty case were gathered. Also, indications for other kinds of corneal tissue specimens were collected with clinicopathological correlation to the final histopathological diagnosis.

### Statistical Analysis

Data was collected on an excel spreadsheet and then transferred to Statistical Package for the Social Sciences (SPSS). For quantitative data, we used the median and interquartile range. The analysis conducted was primarily descriptive in nature. Fisher’s exact was utilized to examine the correlations within our qualitative data. Statistical significance was determined using a p-value threshold of 0.05 or lower.

## Results

### Demographics and Type of Corneal Specimen

A total of 347 corneal tissue specimens were collected from patients ranging in age from 1 day to 89 years, with an average age of 46 ± 21.5 years and a median age of 41 years (IQR = 27.0–67.0). Males accounted for 52.45% of the total specimens, while right eye specimens comprised 46.7% of the samples (Table 1). The corneal tissue specimens were categorized into various types depending on the corneal tissue layers included in the specimen, with the most commonly submitted specimens being full-thickness corneal buttons obtained by PKP (*n* = 172), partial-thickness corneal buttons obtained by LKP (*n* = 75), and DM and endothelium samples obtained mostly from DSAEK. (*n* = 63). These and other less common corneal tissue samples such as biopsies, SK, and removed failed DSAEK grafts are summarized in (Table 2).

**Table 1:** Demographics of patients from whom 5 main types of corneal tissue were obtained and examined histopathologically

Demographic data / specimen	Full thickness cornea (PKP) *n* = 172 (49.6%)	Partial thickness cornea (LKP) *n* = 75 (21.6%)	Superficial keratectomy (SK) & corneal biopsy *n* = 23 (6.6%)	Descemet’s M. and endothelium (DSAEK) *n* = 63 (18.2%)	Removed failed DSAEK flap *n* = 14 (4.0%)	*P* value	Total *n* = 347
Age at presentation in years; Median	39.5	27.0	41.0	69.0	74.0	< 0.001*	41.0
(IQR)	(27.0-61.8)	(26.0–34.0)	(22.0–67.0)	(61.0–78.0)	(65.3–80.3)		(27.0–67.0)
SD	20.6	9.8	24.5	10.8	9.7		21.5
Gender							
Male	95 (55.2)	36 (48.0)	15 (65.2)	32 (50.8)	4 (28.6)	0.207	182 (52.4)
Female	77 (44.8)	39 (52.0)	8 (34.8)	31 (49.2)	10 (71.4)		165 (47.6)
***P*** **value**	0.054	0.625	0.041*	0.858	0.026*		0.206

PKP: Penetrating keratoplasty. LKP: Lamellar keratoplasty. SK: Superficial keratectomy. DSAEK: Descemet’s stripping automated endothelial keratoplasty

*Statistically significant at 5% level of significance

**Table 2:** The number and types of corneal specimens submitted each year

Specimen / year	2015	2016	2017	2018	2019	Total
Full thickness cornea (PKP)	24	36	32	32	48	172
Partial thickness cornea (LKP)	11	12	22	11	19	75
Descemet’s membrane and endothelium (DSAEK)	7	10	12	16	18	63
Corneal biopsy	5	3	2	2	3	15
Failed DSAEK graft	0	4	2	2	6	14
Partial thickness cornea (SK)	0	0	1	2	5	8
**Total**	47	65	71	65	99	347

PKP: Penetrating keratoplasty. LKP: Lamellar keratoplasty. DSAEK: Descemet’s tripping automated endothelial keratoplasty. SK: Superficial keratectomy

Patient demographics stratified by the type of corneal tissue obtained revealed statistically significant differences in the age and gender distributions, as indicated by the interquartile ranges (IQRs), across the different specimen types. The age of patients undergoing PKP or LKP was younger with statistically significant p value when compared to DSAEK procedures with collected DM and endothelium samples (*p < 0.001*). Additionally, there was a statistically significant difference in the age of patients undergoing SK or corneal biopsy with the latter patients being much younger than those who had either DM and endothelium samples obtained at primary DSAEK or failed DSAEK flaps obtained at repeated DSAEK (*p < 0.001 and p < 0.002* correspondingly).

Additionally, gender differences were observed (Table 1), with males being more commonly the source for SK and corneal biopsy specimens (*p = 0.041*), while females were more often the source for failed DSAEK grafts (*p = 0.026*).

The analysis of the indications for the 2 main types of keratoplasty procedures (PKP and LKP) are summarized in Table 3 with keratoconus being the commonest in both (45.3% and 94.7% respectively). Other common indications for PKP included failed previous grafts (16.9%), therapeutic PKP for microbial keratitis (11.0%), PBK or ABK in 8.1% followed by corneal scarring in 7.0%.

**Table 3:** Clinical diagnoses/indication for penetrating keratoplasty (PKP) and lamellar keratoplasty (LKP)

Clinical diagnosis/Surgical indication	Full thickness cornea (PKP) *n* = 172	Partial thickness cornea (LKP) *n* = 75
Keratoconus	78 (45.3%)	71 (94.7%)
Failed PKP/LKP/DSAEK grafts (failed PKP = 21/ failed LKP = 1/ failed DSAEK = 7)	29 (16.9%)	
Microbial keratitis	19 (11.0%)	
Pseudophakic/aphakic bullous keratopathy (PBK/ABK)	14 (8.1%)	
Corneal scarring	12 (7.0%)	2 (2.7%)
Macular Corneal Dystrophy	6 (3.5%)	1 (1.3%)
Noninfectious PUK/exposure keratopathy /Autoimmune corneal perforation	6 (3.5%)	1 (1.3%)
Fuchs’ endothelial dystrophy	3 (1.7%)	
Others: Peters’ anomaly (*n* = 2), post-LASIK ectasia (*n* = 2), and keratoglobus (*n* = 1).	5 (3.0%)	

PKP: Penetrating keratoplasty. LKP: Lamellar keratoplasty. DSAEK: Descemet’s stripping automated endothelial keratoplasty. PUK: Peripheral ulcerative keratitis. PBK: pseudophakic bullous keratopathy. ABK: Aphakic bullous keratopathy

### Corneal Surgical Procedures

**Graph 1:**
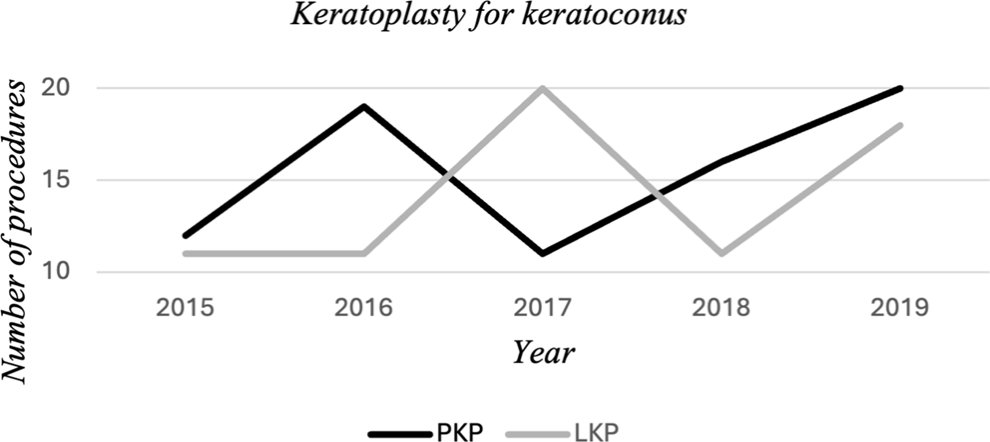
Number of PKPs and LKPs for the treatment of keratoconus

**Graph 2:**
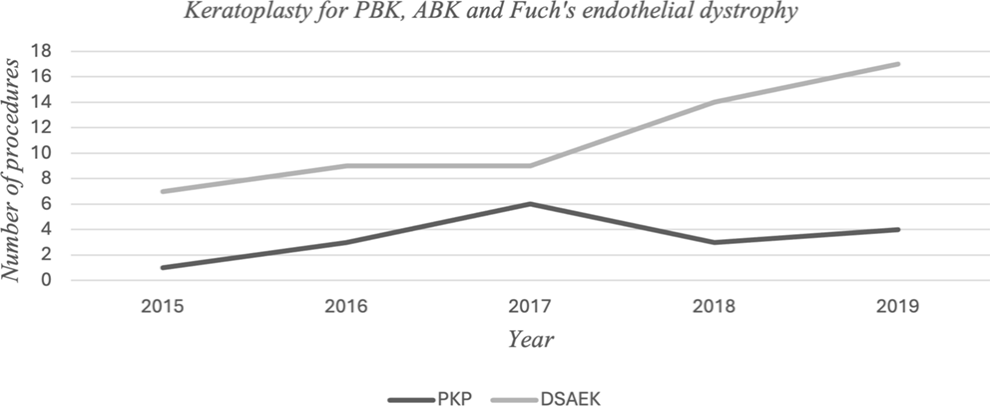
Numbers of PKP and DSAEK performed as treatment for PBK, ABK and FED during the study period with shift of surgical procedure’s preference to DSAEK over the years

The approaches for managing keratoconus by either LKP or PKP has been consistent as shown in Graph 1. However, management options for corneal endothelial decompensation including PBK, ABK and FED has shown a shifting trend from PKP to DSAEK over the years of the study with a statistically significant difference (*p = 0.028*) in the increased percentage of DSAEK between 2015 and 2019, over the total number of procedures done in these 2 above mentioned years- {10/29 = 34.5%} compared to PKP {3/29 = 10.3%} (Graph 2).

Indications for DSAEK representing our main EK procedure during the study period were mostly PBK in 74.6%. The remaining patients had either previously failed PKPs, failed DSAEKs, or corneal decompensation due to endothelial dystrophies (Fuchs or non-guttata) (Table 4). The analysis of the management for 47 patients with failed corneal grafts revealed further nuances in the surgical approaches as summarized in (Table 5).

**Table 4:** The indications for DSAEK procedure with removal of Descemet’s membrane (DM) and the endothelium

Indication for DSAEK procedure	Type of tissue obtained	
	DM & endothelium without posterior corneal stroma *n* = 63	DM & endothelium with posterior corneal stroma (DSEAK flap) *n* = 14
Pseudophakic bullous keratopathy	47 (74.6%)	
Failed DSAEK flap		14 (100%)
Fuchs’ endothelial dystrophy	9 (14.3%)	
Failed PKP graft	4 (6.3%)	
Non-guttata endothelial dystrophy	3 (4.8%)	
**Total**	**63 (100%)**	**14 (100%)**

DSAEK: Descemet’s stripping automated endothelial keratoplasty; DM: Descemet’s membrane; PKP: Penetrating keratoplasty; PBK: Pseudophakic bullous keratopathy

**Table 5:** Management of different types of 47 failed grafts according to the preferred choice of corneal transplant procedure (PKP in 29 patients and DSAEK in 18 patients)

Surgical indication	Failed PKP (*n* = 25)		Failed DSAEK (*n* = 21)		Failed LKP (*n* = 1)
*Management*	Re-PKP *n* = 21 (84%)	DSAEK *n* = 4 (16%)	Re-DSAEK *n* = 14 (66.7%)	PKP *n* = 7 (33.3%)	PKP *n* = 1

PKP: Penetrating keratoplasty. LKP: Lamellar keratoplasty. DSAEK: Descemet’s stripping automated endothelial keratoplasty

The corneal ulceration patients were further categorized into infectious and non-infectious etiologies (Table 6). The infectious group was primarily managed with either corneal biopsy mostly for diagnostic purposes or by definitive therapeutic PKP. In contrast, the non-infectious group was typically treated with either PKP or LKP approaches. Among the infectious group with identified pathogen(s), bacterial keratitis was the most prevalent, followed by fungal, then viral keratitis.

**Table 6:** Different aetiologies for infectious versus non-infectious corneal ulceration cases and their corresponding management

	Aetiology	Full thickness cornea (PKP)	Partial thickness cornea (LKP)	Superficial keratectomy (SK) & corneal biopsy	Total *n* = 33
Infectious	Bacterial	7	0	6	13
	Fungal	7	0	0	7
	Viral-Herpetic	2	0	0	2
	Unknown Organism	3	0	1	4
Non-infectious	Exposure keratopathy	3	0	0	3
	PUK	2	1	0	3
	Autoimmune disease	1	0	0	1
**Total**		**25**	**1**	**7**	**33**

PKP: Penetrating keratoplasty. LKP: Lamellar keratoplasty. SK: Superficial keratectomy. PUK: Peripheral ulcerative keratitis

## Discussion

Most of the literature on keratoplasty procedures has been focused on the clinical analysis, surgical procedures, and outcomes for patients who undergo such procedures, with few studies evaluating the histopathological aspects of the case. Our retrospective study aimed to present the demographics, surgical indication trends, and histopathological findings of all corneal specimens submitted to our tertiary eye care hospital laboratory for tissue diagnosis over 5 years. Most of our specimens were obtained from variable keratoplasty procedures namely PKP, LKP, and DSAEK. Other sources included smaller numbers of SKs, corneal biopsies and DSEAK grafts.

In this discussion, we will focus on the leading clinical conditions and the variability of the demographics for the most commonly performed keratoplasty procedures in our hospital and delineate and identify any surgical trends found.

Keratoconus is the leading indication for corneal transplantation globally. A systematic review and meta-analysis revealed that Saudi Arabia has the highest reported prevalence of keratoconus among the 15 countries examined [5]. Even though this study is histopathological-based and not clinically- based, therefore, cases of keratoconus that are managed conservatively without a corneal tissue submitted are not represented but since the study is conducted in a tertiary care eye center, the prevalence of keratoconus among patients undergoing PKP/LKP is truly reflecting the high frequency of KC among these surgical cases in total. This aligns with findings from multiple studies conducted within Saudi Arabia over the past several years, as well as the findings in our study (Table 3) with keratoconus being the commonest indication for about 95% of our LKP and almost half of our PKP [6–9]. Studies from other regions have also identified keratoconus as a primary indication for corneal transplantation. For example, investigations in Italy and Iran have reported keratoconus as the most common reason for corneal grafting procedures [10, 11]. In contrast, the American population exhibited the lowest reported prevalence of keratoconus across the countries surveyed [5]. The high prevalence of keratoconus in Saudi Arabia may be partially attributed to the demographic profile of the population, with 36.2% of individuals between 15 and 34 years of age [12]. Keratoconus often manifests in younger patients, making this age distribution a contributing factor [13]. This was evident in our study with a statistically significant difference in the age of patients undergoing PKP or LKP compared to DSAEK procedures (younger in the earlier 2 procedures with *p* < 0.001). This observed disparity is likely attributable to the predilection of corneal ectasia, the primary indication for PKP and LKP, to occur in younger patient populations [13]. It is also worth mentioning that there was no statistically significant gender distribution in this group of patients (Table 1). The histopathological features of the corneal tissue obtained from keratoconus patients in our study demonstrated what are considered to be hallmarks of keratoconus with defects in Bowman’s layer, wavy deformity of the collagenous lamellae, and generalized stromal thinning with or without significant scarring [14]. Despite the availability of newer surgical techniques, such as intra-stromal corneal ring segments (ICRS) insertion, cross-linking, and variety of LKP, which have demonstrated improved visual outcomes and graft survival rates compared to traditional PKP in keratoconus cases [15, 16], the study data did not show a consistent shift towards greater adoption of LKP over the 5-year period examined (Graph 1). This is likely because in advanced keratoconus with severe central corneal thinning, deep scarring, prior hydrops, complicated or failed ICRS, and intraoperative DM rupture, PKP is often preferred or becomes the necessary surgical approach [15, 17]. This trend in terms of the choice of the surgical procedure is also applicable in our institution where PKP is reserved for advanced KC cases with severe thinning especially following resolved acute hydrops. It is important to note that LKP is a technically more challenging procedure compared to PKP [18], which may also contribute to the fluctuating surgical trend in our hospital observed in graph 1 over the years, with persisting prominence of PKP as a management option for keratoconus.

Previously failed corneal grafts were the second most common indication for PKP in about 17% of all full-thickness corneal specimens examined (Table 3). This finding is slightly higher than the results of a previous 10-year study conducted at our institution (KAUH), in which regrafting was the third most common indication, comprising 10% of all corneal transplantations [6]. In the present study, the growing number of regrafts coincides with the overall increase in corneal transplantation procedures. It is worth mentioning that a small number of the failed PKP cases 4/25 (16%) were managed by DSAEK. This choice in the surgical procedure is guided by the general condition of the anterior part of the cornea where PKP is favored in cases with sub-epithelial fibrosis and marked corneal stromal scarring. While DSAEK is reserved for the smaller number of cases with relatively healthy corneal tissue anteriorly. On the other hand, about two thirds of the total 21 failed DSAEK grafts were managed by repeated DSAEK procedures (66.7%) compared to one third only undergoing PKP (Table 5).

Other less common indications for PKP included microbial keratitis (for which it was utilized as a therapeutic intervention), corneal decompensation conditions (PBK, ABK, and FED), corneal scarring, and corneal dystrophies/degenerations (Table 3). Macular dystrophy was the sole corneal stromal dystrophy observed in the current cohort. Histopathological analysis of 6 full-thickness corneal buttons and 1 partial-thickness specimen demonstrated stromal deposition that stained positively with alcian blue and colloidal iron, along with irregularities in the epithelial and Bowman’s layers. These findings were consistent with the characteristic pathological features of macular corneal dystrophy [19]. Interestingly, a 1991 study conducted in Saudi Arabia reported macular dystrophy to be the most common corneal dystrophy, accounting for 62% of all corneal dystrophies specimens [20]. A more recent analysis of corneal stromal dystrophies presenting to a tertiary eye hospital in Saudi Arabia between 2002 and 2011 have found macular dystrophy to be the predominant subtype, comprising 93.26% of all stromal dystrophy requiring keratoplasty [19]. In contrast, granular dystrophy has been more frequently reported in other geographical regions, such as Japan [21], Europe, and the United States [22].

Postoperative bullous keratopathy is a common indication for keratoplasty procedures with a reported prevalence of 14.8% in a previously published data from our institution prior to 2015 [6]. It is expected that bullous keratopathy may be declining over time due to recent improvements in phacoemulsification techniques and newer intraocular lens designs, which have reduced the incidence of corneal decompensation following cataract extraction [23, 24]. A review study found a decreasing prevalence of PBK and ABK from 19.4% in 1980 to 16.7% in 2001 [25]. Patients presenting with PBK (*n* = 60) and one ABK patient comprised a significant proportion of our total corneal transplant specimens with a history of intraocular surgery and endothelial attenuation on histopathological examination. Out of this cohort, 47 patients with PBK were managed with the DSAEK procedure and this diagnosis was the commonest indication (in about 75%) for DSAEK as per Table 4, while 14 patients, including the ABK patient, underwent PKP. Full-thickness corneal grafting was previously considered the gold standard for these cases as well as for other causes of corneal decompensation, but EK techniques are now preferred due to lower rejection risk, better visual outcomes, and faster recovery [18, 26]. The DMEK procedure wasn’t yet adopted and mastered at our institution during the study period, therefore, DSAEK was the sole EK technique used.

In the present study, FED was a recognized cause for corneal decompensation in 12 patients, out of whom 9 underwent DSAEK, while 3 had PKP. Histopathological examination revealed features characteristic of FED, including irregular and thickened DM, well-formed guttata, and markedly attenuated endothelium [27] On the other hand, 3 patients underwent DSAEK procedure (Table 4) but the submitted DM didn’t show any guttata. A previous study published in Saudi Arabia also identified FED as the most common corneal endothelial dystrophy [20]. EK techniques have shown high success rates in managing FED, thereby reducing the need for traditional PKP [28]. The trend for managing corneal decompensation surgically has significantly changed over time in our institution with the evolving DSAEK techniques becoming the preferred surgical technique over PKP, as shown in graph 2.

Regarding failed grafts, most of the failed PKP and LKP patients were managed with repeated PKP as described above, while most of the failed DSAEK patients were treated with repeated DSAEK procedure (Table 5). The received DSAEK flaps in these showed variable attenuation of the donor endothelium. An incidental, statistically significant finding was that females were more commonly the source of failed DSAEK flaps (*p = 0.026*) compared to males (Table 1). The present analysis also revealed statistically significant differences (*p < 0.001*) in the median ages of patients undergoing PKP/LKP with full/partial-thickness corneal specimens obtained, compared to those in whom DM and endothelium specimens were obtained via DSAEK procedure (Table 1). Corneal decompensation due to any etiology mentioned above and failure of previous grafts are expected to occur at a later age, which explains this age predilection in DSAEK specimens of our study cohort [29].

Corneal biopsy has a diagnostic role in microbial keratitis and is suggested in cases of falsely negative results of corneal scrape, refractory cases to medical treatment, or deeply situated infiltrate that are not amenable to scrape [30]. While PKP serves as a diagnostic and therapeutic procedure, therapeutic PKP is performed in cases of deterioration despite maximal therapy, descemetocele formation, scleral involvement, threat to globe integrity or perforation [31]. Notably, in our cohort, SK and corneal biopsy were more frequently performed on male patients compared to their female counterparts. This observed disparity may be attributed to the increased likelihood of males sustaining occupational-related trauma, exposure to environmental hazards during outdoor activities, and infections. These factors may elevate their susceptibility to developing degenerative corneal conditions and microbial keratitis. Numerous studies have corroborated this trend in gender-based differences [32–34].

## Conclusion

In conclusion, the trends in keratoplasty have undergone continuous evolution. The data suggests that keratoconus remains the most common indication for LKP cases, and for less than half of the PKP procedures. Corneal decompensation, either post cataract surgery, failed previous graft, or secondary to endothelial dystrophy has been a common indication for PKP and DSAEK, and there has been a significant shift from PKP to DSAEK in managing these conditions. PKP and LKP were more commonly indicated in younger patients in relation to the underlying common corneal disease, whereas DSAEK was predominantly performed in older age groups. There was a male gender predilection in patients undergoing SK and diagnostic corneal biopsy, potentially due to the lifestyle in our country with the males being more exposed to outdoor activities, environmental factors, and occupational hazards. These findings highlight the specific need for surgical expertise in our country based on the higher prevalence of keratoconus and the common current practice. We need to address the necessity for advanced training of cornea surgeons to meet the evolving landscape of corneal transplantation with tailored surgical approaches according to the risk profiles of different patient populations.

## Data Availability

The datasets used and analyzed during the current study are available from the corresponding author upon reasonable request.

## Abbreviations

PKP: Penetrating keratoplasty
LKP: Lamellar keratoplasty
DSAEK: Descemet’s stripping automated endothelial keratoplasty
SK: Superficial keratectomy
DM: Descemet’s membrane
ICRS: Intrastromal corneal ring segments
PUK: Peripheral ulcerative keratitis
PBK: Pseudophakic bullous keratopathy
ABK: Aphakic bullous keratopathy
FED: Fuch’s endothelial dystrophy
EK: Endothelial keratoplasty
